# Longitudinal Clinical Debrief in an Undergraduate Medical Programme

**DOI:** 10.1111/tct.70494

**Published:** 2026-08-10

**Authors:** Susan Harris, Aaron Drovandi, Shireen Tahir, Sarah Merrifield

**Affiliations:** ^1^ Division of Medical Education, School of Medical Sciences University of Manchester Manchester UK

**Keywords:** clinical reasoning, community of practice, mixed‐methods, safe space

## Abstract

**Background:**

Explicit teaching of clinical reasoning to medical students can be difficult to achieve, though may be facilitated through structured clinical debrief sessions supporting student discussions of case studies and placement experiences. This study aimed to understand student perceptions of the value of clinical debrief throughout their curriculum.

**Approach:**

A scaffolded series of clinical debrief sessions were incorporated into Years 1, 3 and 5 of a 5‐year UK undergraduate medical programme. Sessions are increasingly complex across year‐levels and continuously improved across academic years according to student feedback. Each session receives student feedback through Likert‐scale and open‐ended questions on the session and their facilitating tutor, which were consolidated and analysed using descriptive statistics and AI‐assisted conceptual content analysis.

**Evaluation:**

From over 8000 responses across the three year‐levels and nine academic years, clinical debrief was positively viewed across all cohorts, with increasing perceived value for Year 3 students, though some decrease in perceptions for Year 5 students. Earlier year students emphasised the value for their communication and clinical reasoning skills, while later year students valued the ethical and medico‐legal skill development and preparing for practice as a doctor. Tutors across all year levels were perceived highly for their contributions to an inclusive and effective learning environment and reducing professional isolation.

**Implications:**

Scaffolded clinical debrief is positively viewed by undergraduate medical students across year levels, with perceived contributions to skill development influenced by year level. Development and implementation of clinical debrief require design aligning to student experiences and expectations, with effective tutor training.

## 1 Background

Clinical reasoning is a complex cognitive process underpinning clinical decision making, defined as the gathering and interpretation of patient data to formulate diagnostic and treatment plans [1, 2]. Effective clinical reasoning skills are crucial to providing safe, quality patient care such as reduced misdiagnosis and inappropriate treatment [2, 3]. Within medical training, clinical reasoning development is traditionally considered a by‐product of clinical rotations and placement [4]. More recently, explicit integration of clinical reasoning has been recognised as part of effective longitudinal spiral curricula [2]. Best practice for clinical reasoning teaching is non‐specific with no single definition or agreed method [5]. However, general domains have been identified, including gathering data from history and examination, diagnostic tests, differential diagnoses and management and shared decision making [5, 6]. Enquiry‐based and practical learning with serial cues is more effective than teaching the theoretical basis, with longitudinal rather than cross‐sectional approaches superior for student learning [3, 6].

A recent systematic review highlighted data on the effectiveness of clinical reasoning teaching methods within undergraduate curricula is fragmented, with a notable gap in interventions that examine student progression and outcomes in clinical reasoning longitudinally [5]. There are also few interventions for clinical reasoning that have been implemented in the pre‐clinical years, as well as a lack of understanding of how students value clinical reasoning as an explicit element of their curriculum [5]. Once such intervention is ‘Clinical Debrief’, a structured, small‐group discussion intended to support student well‐being and development of clinical reasoning skills through exploration of clinical experiences [7]. This study aimed to explore undergraduate medical student perceptions and perceived value of clinical debrief teaching when used longitudinally across a curriculum. This study employed recurring anonymous online surveys with Year 1, 3 and 5 undergraduate students being provided ‘Clinical Debrief teaching’ at the University of Manchester. As the data used in this study originated from routine evaluation, ethical exemption was approved by the University of Manchester Proportionate Research Ethics Committee (2024‐21408‐37716).

## 2 Approach

### 2.1 Local Context

**FIGURE 1:**
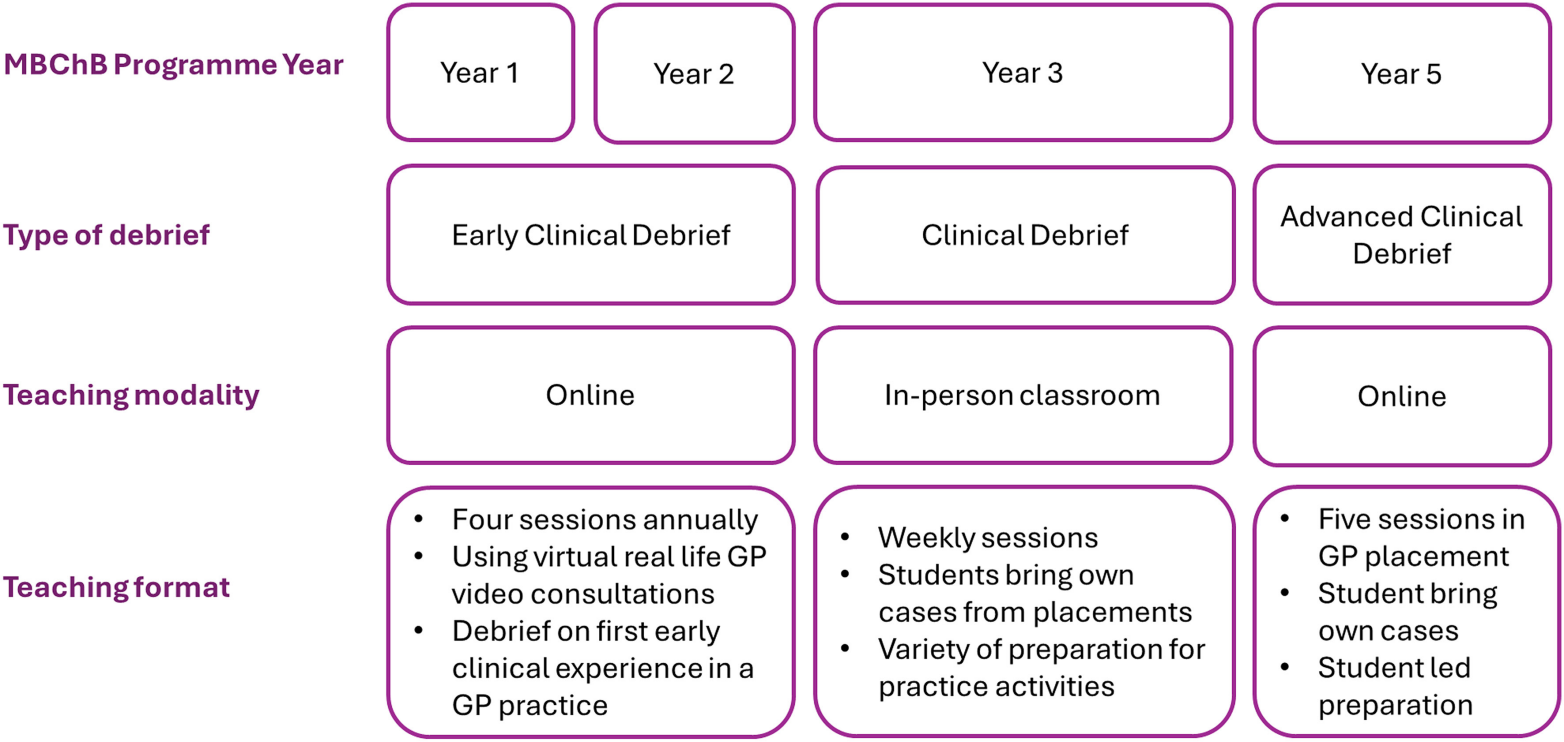
Longitudinal clinical debrief within the University of Manchester undergraduate Medical Programme.

The University of Manchester undergraduate MBChB has a longitudinal strategy to teach clinical reasoning, with the ‘Clinical Debrief’ element using the Clinical Reasoning Tool [2] introduced in 2016 for Year 3 medical students as they transitioned to their clinical years [7]. Success led to expansion to ‘Early Clinical Debrief’ for pre‐clinical and ‘Advanced Clinical Debrief’ for final‐year medical students, providing this opportunity to examine the perceived value of clinical debrief longitudinally across pre‐clinical, clinical and final year levels. Figure 1 illustrates these sessions throughout the programme. Themes across years focus on clinical experiences (safe space to share emotional experiences and promote wellbeing), clinical reasoning (via case discussion), clinical practice preparation, role modelling, and building professional identities [2, 7, 8]. A scaffolded approach uses curated consultations, at appropriate complexity for each year level. The approach utilises an open culture with psychological safety, and recognition that learners need to feel comfortable to allow the depth of discussion that yields critical thinking and reflection [7, 8].

### 2.2 Participants and Data Collection

During (or shortly after) the final session of each semester, students are invited to complete a survey on their Clinical Debrief Sessions, containing 5‐point Likert‐scale questions and open‐ended questions on what they found beneficial and what required improvement. Likert‐scale questions ask about meeting learning needs, developing clinical reasoning skills, developing professionalism and practicing ethically, receiving constructive feedback, improving learning while on placement, supportive learning environment and overall experience. For each question, students could rate strongly disagree, disagree, neutral, agree or strongly agree. The survey was developed by Clinical Debrief team, a group of clinically active General Practitioner academics [7]. The survey is distributed through ‘eForms’, an online system that allows university staff to build a broad range of data collection tools. All questions are displayed in a standardised order on a single page. The evaluation survey is not mandatory, and there are no incentives for students. The survey templates for each year level are available in Appendix S1.

### 2.3 Data Analysis

Likert‐scale data were summarised narratively using SPSS v26 (IBM Corp, Armonk, NY, USA). Core questions used consistently across years were incorporated into line graphs to demonstrate student perceptions trends over time and trends in perceptions as they advanced through the programme. Given the volume of open‐ended responses collected (> 8000), traditional content analysis was not feasible. Author SH therefore developed and utilised a structured set of CoPilot prompts to perform a conceptual content analysis [9] to facilitate comparison of emerging themes to the Likert‐scale questions [10, 11]. Prior to input into CoPilot, comments were pre‐screened to remove individual‐identifying data. Examination of CoPilot responses and decision processes necessitated five iterations before the final prompt was agreed and used (Appendix S2). An element of these prompts was a chronological approach similar to the Likert‐scale questions, with thematic trends considered at each academic year. Authors with roles in the Clinical Debrief team (SH, SM) discussed and confirmed the developed themes aligned with their experiences and annual reviews of student feedback. Illustrative quotes that demonstrate the key themes identified are reported verbatim as exemplars.

## 3 Evaluation

Since inception of Clinical Debrief for Year 3 students in the 2016/2017 academic year to the 2024/2025 academic year for Year 1, 3 and 5 students, there were 8126 student responses collected; 1412 for Year 1, 6023 for Year 3 and 691 for Year 5 (Appendix S3). The survey was anonymous, meaning it was not possible to collect student characteristics or link their characteristics to Likert‐scale or open‐text responses. Average scores to the Likert‐scale questions are included in Appendix S1, indicating positive perceptions across all academic years and year levels. Early Clinical Debrief had insufficient consistent questions to examine trends, though for Clinical Debrief there was a consistent upwards trend across academic years (apart from tutor approachability which remained steady), and for Advanced Clinical Debrief, there was a downwards trend across academic years.

The four strongest thematic trends across year levels and academic years related to perceived value in developing communication and clinical reasoning skills, developing professional values and ethical and medico‐legal skills, tutor capacity to build inclusive and effective learning environments and logistical and administrative challenges. Skill development was emphasised across cohorts, though students valued specific skill growth according to their year level. Year 1 students found clinical debrief beneficial for development of initial medical communication skills (21% of respondents), which in Year 3 was reduced in emphasis and replaced by clinical reasoning development (18% of respondents), remaining high in Year 5 (18% of respondents) with further specific emphasis on data interpretation, prescribing skills and ethical and medico‐legal considerations within medical practice.

Gave us lots of time to practice and discuss our own communication skills and our thoughts on the observed skills within the example GP session. (Y1)

Our sessions really improved my presentation of patient assessments as well as clinical reasoning, forming differential diagnosis' and creating management plans. (Y5)

Skill development was perceived to be tied to tutor capacity to facilitate a learning environment which felt safe to share experiences, concerns and questions. Apart from tutor approachability and enthusiasm, their sharing of lived experiences and promoting reflective sharing were considered key elements of an effective clinical debrief tutor.

Engaging, friendly, great listener, allowed us to have a say in the content, covered useful subjects, involved the whole group and gave us all equal opportunity to participate. (Y3)

The key limitations or requested improvements of the clinical debrief element of the programme were logistical or administrative in nature, such as scheduling limitations, technical issues, sessions that felt repetitive of discussions held on placement, and group size.

We did it in year 3 and I don't feel there was much more to gain … Discussing two cases every week just gets repetitive, the tutor does her best to make it interesting. (Y5)

## 4 Implications

This study examined undergraduate medical student perceptions of the value of scaffolded clinical debrief embedded across 5 years of a medical programme. Despite some differences in specific questions across year levels, there were consistent findings relating to perceived skill development and tutor contributions to the learning environment. Promoting medical student awareness of their acquisition of less tangible skills such as clinical reasoning is an inherently difficult though essential role of health professional educators [5]. It is not feasible to teach clinical reasoning in a single session or through a single type of experience such as placement, with the scaffolded clinical debrief approach described here proposed to be a valuable partner to clinical placement experiences according to students' reception to this element of their teaching [6]. As student knowledge and experiences grow through their educational journey, the focus of elements of teaching activities such as clinical debrief also needs to grow and focus on the skills that students both need and perceive that they need [12].

*As student knowledge and experiences grow through their educational journey, the focus of elements of teaching activities such as clinical debrief also needs to grow and focus on the skills that students both need and perceive that they need*.

Further to the complexities surrounding what should be taught and when is the considerations on who delivers teaching and how. Apart from content knowledge and enthusiasm for teaching [13, 14], for students to be able to raise and discuss clinically, ethically or morally complex cases, an effective educator needs to create an environment capable of hosting these discussions [15]. Psychologically safe spaces need to be built and maintained by educators, with this perceived safety fluctuating according to members within the room, discussions being held and pre‐existing biases and beliefs held by these stakeholders [15].

Readers should consider the strengths and limitations of this research when interpreting the findings and applying them within their own contexts. Strengths include the volume of data achieved through the large number and generally high proportion of respondents, and use of consistent question types across year levels and academic years. There are also several limitations, including the lack of capturing student characteristics and availability of analyses according to these characteristics, which may have provided further insights into how different student populations benefit from clinical debrief. There is also variability in response rates within and across year levels, introducing a risk of participation bias where student responses do not reflect true perceptions across the wider cohort. There is also a restriction of data to student perceptions (Level one of the Kirkpatrick scale) [16], and finally, the use of an AI tool to support content analysis invites risking misinterpretation of the broader dataset. However, the authors believe that this risk has been effectively mitigated through a combination of long‐standing coordination of Clinical Debrief by the first author providing regular insights into data trends and use of an iterative process in crafting AI prompts.

## Author Contributions

**Susan Harris:** conceptualization, data curation, formal analysis, writing – review and editing. **Aaron Drovandi:** formal analysis, investigation, methodology, supervision, writing – original draft. **Shireen Tahir:** formal analysis, visualization, writing – review and editing. **Sarah Merrifield:** conceptualization, data curation, writing – review and editing.

## Funding

The authors have nothing to report.

## Conflicts of Interest

All authors are academics with teaching roles within the undergraduate medical degree at the University of Manchester.

## Supporting information


**Appendix S1:** Survey questions per year level per academic year.
**Appendix S2:** Development of CoPilot prompts for analysis of free‐text responses.
**Appendix S3:** Number of responses for year level per academic year.

## Data Availability

Data are available from the corresponding author on reasonable request.
