# Supplementary material for: Longitudinal Clinical Debrief in an Undergraduate Medical Programme
Source: Clin Teach. 2026 Aug 10;23(5):e70494. doi: 10.1111/tct.70494 (PMC13457512; doi:10.1111/tct.70494)
Supplement: Supplementary file 1 [file TCT-23-e70494-s001.docx]

# Appendix 1: Survey questions per year level per academic year

**Early Clinical Debrief (Year 1) –** 2021/2022 to 2024/2025 academic years (4 years)

Likert-scale questions – organised chronologically by academic year introduced

| Question* | 2021 (224) | 2022 (112) | 2023 (861) | 2024 (215) |
| --- | --- | --- | --- | --- |
| During the debrief session I had the opportunity to reflect on my ECE visit experience, or the alternative virtual primary care experience if I was unable to go on an ECE visit. | 4.44  [4.35-4.53] | 4.24  [4.05-4.43] | - | - |
| The debrief session increased my understanding of how health and social care works in the community. | 3.79  [3.66-3.91] | - | - | - |
| In the debrief session, I was able to discuss challenging or educationally interesting experiences. | 4.26  [4.16-4.37] | 4.26  [4.06-4.45] | - | - |
| The session was useful, in that I was able to hear about other students’ experiences on placement. | 4.30  [4.18-4.43] | 4.13  [3.94-4.33] | - | - |
| I was given the opportunity to discuss any uncertainties from my visit. | 4.09  [3.97-4.21] | 3.95  [3.75-4.14] | 4.69  [4.64-4.73] | 4.82  [4.76-4.89] |
| Overall, the debrief session was useful for my learning. | 3.79  3.65-3.93] | 3.58  [3.38-3.78] | 4.37  [4.31-4.42] | - |
| The sessions improved my communication skills | - | - | 4.06  [4.00-4.12] | 4.54  [4.45-4.63] |
| The session enhanced my understanding of clinical reasoning | - | - | 4.30  [4.24-4.35] | 4.52  [4.43-4.60] |
| My tutor supported the development of my clinical reasoning skills. | - | - | - | 4.74  [4.67-4.82] |
| My tutor effectively facilitated discussions around ethical and professional issues. | - | - | - | 4.78  [4.71-4.85] |
| My tutor gave constructive feedback that has/will improve my learning. | - | - | - | 4.52  [4.42-4.62] |
| My tutor was approachable and easy to communicate with. | - | - | - | 4.90  [4.85-4.95] |
| The sessions provided a supportive space where I could discuss any challenges from medical school or clinical placements | - | - | - | 4.71  [4.63-4.79] |

* 5-point scale; significant improvement needed, needs improvement, acceptable, good, excellent. Others on a 5-point scale; Strongly Disagree, Disagree, Neutral, Agree, Strongly Agree. Numbers in round brackets is the responses collected for that academic year. Numbers in square brackets are the 95% confidence intervals for each mean value.

Open-ended questions with free-text responses (each used for all 4 years):

1. What elements of the sessions do you think were useful for you?
2. What elements of the sessions do you think were missing or could have been improved?
3. Is there any other feedback that you would like to give to support us in improving these sessions

**Clinical Debrief (Year 3)** – 2016/2017 to 2024/2025 academic years (9 years)

| Question* | 2016 (547) | 2017 (642) | 2018 (666) | 2019 (649) | 2020 (647) | 2021 (550) | 2022 (747) | 2023 (837) | 2024 (738) |
| --- | --- | --- | --- | --- | --- | --- | --- | --- | --- |
| My tutor helped me identify my learning needs | 4.35  [4.29-4.41] | 4.44  [4.39-4.49] | 4.41  [4.36-4.47] | 4.56  [4.52-4.61] | 4.51  [4.46-4.56] | 4.56  [4.50-4.61] | 4.65  [4.61-4.69] | 4.63  [4.59-4.67] | 4.61  [4.56-4.65] |
| My tutor was effective at facilitating development of my clinical reasoning skills (formulating differential diagnoses) | 4.54  [4.49-4.60] | 4.59  [4.54-4.63] | 4.58  [4.53-4.63] | 4.76  [4.72-4.79] | 4.69  [4.65-4.73] | 4.74  [4.70-4.78] | 4.77  [4.74-4.81] | 4.78  [4.74-4.81] | 4.76  [4.73-4.80] |
| My tutor was effective at facilitating discussions around ethical and/or professionalism issues | 4.50  [4.44-4.56] | 4.54  [4.48-4.59] | 4.53  [4.48-4.58] | 4.71  [4.66-4.75] | 4.63  [4.58-4.67] | 4.70  [4.66-4.75] | 4.78  [4.74-4.82] | 4.73  [4.69-4.77] | 4.74  [4.70-4.78] |
| My tutor gave constructive feedback | 4.43  [4.38-4.49] | 4.48  [4.43-4.53] | 4.48  [4.42-4.53] | 4.62  [4.57-4.66] | 4.56  [4.51-4.61] | 4.62  [4.57-4.67] | 4.68  [4.64-4.73] | 4.63  [4.59-4.67] | 4.85  [4.82-4.88] |
| My tutor was approachable | 4.76  [4.71-4.80] | 4.75  [4.70-4.79] | 4.72  [4.67-4.76] | 4.78  [4.74-4.82] | 4.78  [4.73-4.82] | 4.85  [4.81-4.88] | 4.80  [4.77-4.84] | 4.77  [4.73-4.80] | 4.77  [4.73-4.81] |
| The case discussions were useful in developing my clinical reasoning skills | 4.47  [4.40-4.52] | 4.51  [4.45-4.56] | 4.51  [4.46-4.56] | 4.66  [4.61-4.70] | 4.62  [4.57-4.66] | 4.58  [4.52-4.63] | 4.70  [4.66-4.75] | 4.70  [4.66-4.74] | 4.71  [4.67-4.75] |
| Discussions in Clinical Debrief supported me to optimise learning opportunities on my placement | 4.19  [4.12-4.27] | 4.27  [4.20-4.33] | 4.23  [4.16-4.30] | 4.48  [4.42-4.53] | 4.36  [4.30-4.42] | 4.43  [4.36-4.49] | 4.59  [4.54-4.63] | 4.54  [4.49-4.59] | 4.60  [4.56-4.65] |
| Clinical Debrief has helped me explore my personal & professional values and judgements | 4.24  [4.17-4.31] | 4.33  [4.26-4.38] | 4.27  [4.21-4.33] | 4.53  [4.47-4.58] | 4.40  [4.34-4.46] | 4.38  [4.32-4.45] | 4.61  [4.57-4.66] | 4.54  [4.50-4.59] | 4.59  [4.55-4.64] |
| Clinical Debrief was a supportive environment to learn and reflect in | 4.53  [4.47-4.58] | 4.55  [4.49-4.60] | 4.55  [4.50-4.61] | 4.68  [4.64-4.73] | 4.63  [4.58-4.67] | 4.67  [4.62-4.71] | 4.77  [4.73-4.81] | 4.71  [4.67-4.75] | 4.76  [4.73-4.80] |
| The group gave constructive feedback | 4.12  [4.05-4.19] | 4.28  [4.21-4.33] | 4.23  [4.17-4.30] | 4.46  [4.41-4.52] | 4.42  [4.37-4.47] | 4.43  [4.37-4.49] | 4.63  [4.59-4.67] | 4.54  [4.49-4.58] | 4.62  [4.58-4.66] |
| Overall I would rate my Clinical Debrief sessions as* | 4.23  [4.12-4.30] | 4.31  [4.25-4.38] | 4.31  [4.25-4.35] | 4.46  [4.40-4.50] | 4.33  [4.26-4.38] | 4.34  [4.27-4.40] | 4.53  [4.48-4.57] | 4.47  [4.41-4.51]] | 4.70  [4.65-4.74] |

* 5-point scale; significant improvement needed, needs improvement, acceptable, good, excellent. Others on a 5-point scale; Strongly Disagree, Disagree, Neutral, Agree, Strongly Agree. Numbers in round brackets is the responses collected for that academic year. Numbers in square brackets are the 95% confidence intervals for each mean value.

Open-ended prompts with free-text responses (each used for all 9 years):

1. Please give a highlight of your clinical debrief sessions (This may include aspects relating to the structure and content of your sessions).
2. Please suggest a way in which Clinical Debrief could be improved.
3. Please give a highlight of your clinical debrief sessions (Related to your Tutor/Teaching).
4. Please suggest a way in which you feel your Clinical Debrief tutor could have improved your sessions.

**Advanced Clinical Debrief (Year 5) –** 2021/2022 to 2024/2025 academic years (4 years)

Likert-scale questions – organised chronologically by academic year introduced

| Question* | 2021 (298) | 2022 (205) | 2023 (70) | 2024 (118) |
| --- | --- | --- | --- | --- |
| My lecturer helped me to optimise my learning from my GP placement | 4.64  [4.57-4.71] | 4.71  [4.64-4.78] | 4.64  [4.48-4.81] | 4.42  [4.25-4.48] |
| My lecturer helped me to apply a structured approach to generate a differential diagnosis from the case presentation each week | 4.73  [4.67-4.79] | 4.75  [4.68-4.82] | 4.71  [4.59-4.84] | 4.51  [4.37-4.65] |
| My lecturer helped me to appraise the full range of information gathered about a patient to propose an appropriate management plan | 4.72  [4.66-4.78] | 4.75  [4.69-4.82] | 4.76  [4.64-4.88] | 4.52  [4.38-4.66] |
| My lecturer facilitated discussions around the ethical and/or legal issues arising from our experiences in General Practice | 4.77  [4.71-4.83] | 4.75  [4.68-4.82] | 4.74  [4.62-4.86] | 4.56  [4.43-4.69] |
| My lecturer was effective at facilitating discussions around the impact of my placement on my wellbeing | 4.73  [4.67-4.80] | 4.68  [4.60-4.76] | 4.76  [4.63-4.89] | 4.50  [4.34-4.66] |
| Constructive feedback from my lecturer enabled me to improve my clinical practice during my placement | 4.58  [4.50-4.65] | 4.60  [4.51-4.69] | 4.61  [4.47-4.76] | 4.29  [4.12-4.46] |
| My ACD lecturer facilitated an inclusive learning environment to enable me to feel comfortable in participating and sharing my opinions/ values. | 4.82  [4.76-4.87] | 4.80  [4.72-4.87] | 4.80  [4.68-4.92] | 4.66  [4.54-4.78] |
| Discussions in Advanced Clinical Debrief helped me to develop skills for shared decision making with patients (including their representatives/carers) | 4.48  [4.40-4.56] | 4.51  [4.41-4.61] | 4.61  [4.47-4.76] | 4.26  [4.09-4.44] |
| Discussions in Advanced Clinical Debrief helped me to consider the patient’s personal, cultural and social context in their management | 4.49  [4.40-4.58] | 4.47  [4.37-4.58] | 4.61  [4.47-4.76] | 4.27  [4.09-4.45] |
| Advanced Clinical Debrief supported me to develop the skills for summarising and presenting patient assessments to colleagues | 4.50  [4.41-4.58] | 4.52  [4.41-4.62] | 4.63  [4.49-4.77] | 4.20  [4.01-4.39] |
| Discussions in Advanced Clinical Debrief helped me to apply clinical reasoning skills to identify and manage uncertainty | 4.64  [4.58-4.71] | 4.55  [4.45-4.64] | 4.60  [4.45-4.75] | 4.42  [4.26-4.57] |
| ACD helped me to consider how my experiences in placement could prepare me for what is expected of me as a foundation doctor. | 4.53  [4.46-4.61] | 4.45  [4.35-4.53] | 4.54  [4.38-4.70] | 4.31  [4.14-4.49] |
| Advanced Clinical Debrief has helped support my personal and professional development as a future doctor | 4.52  [4.46-4.61] | 4.47  [4.36-4.57] | 4.47  [4.29-4.65] | 4.30  [4.12-4.47] |
| Advanced Clinical Debrief was a supportive environment in which to learn and reflect in | 4.71  [4.65-4.78] | 4.67  [4.59-4.75] | 4.77  [4.66-4.89] | 4.58  [4.44-4.71] |
| Overall I would rate my Advanced Clinical Debrief sessions as | 4.28  [4.18-4.37] | 4.33  [4.22-4.40] | 4.27  [4.07-4.46] | 4.09  [3.86-4.21] |

* 5-point scale; significant improvement needed, needs improvement, acceptable, good, excellent. Others on a 5-point scale; Strongly Disagree, Disagree, Neutral, Agree, Strongly Agree. Numbers in round brackets is the responses collected for that academic year. Numbers in square brackets are the 95% confidence intervals for each mean value.

Open-ended prompts with free-text responses (each used for all 4 years):

1. Please give up to 3 examples of the most useful aspects of the advanced clinical debrief sessions.
2. Please suggest up to 3 ways in which Advanced Clinical Debrief could be improved.

# Appendix 2: Development of CoPilot Prompts for Analysis of Free-text Responses

The outputs of each iterative prompt detailed below were reviewed and discussed between the two first authors, including an examination of the processing of the data described by the AI tool. Where uncertainty was raised in the processing of the data, subsequent prompts were targeted at these uncertainties, until the authors were satisfied. Where specific data points were requested, such as extracting quotes, these quotes were manually searched by the authors to confirm no specific data point was hallucinated by the AI tool.

## Clinical Debrief (MBChB Year 3, largest data set and first to be analysed) January – March 2026

### Prompt v1

“The spreadsheet attached shows anonymised student course evaluation comments for a 3rd Year undergraduate MBChB teaching session in the UK. Each academic year given its own tab. There are two columns - A is Positive feedback and B is Areas for Development. Please can you analyse the feedback to see if there are any over-arching trends or themes. Please can you advise if the themes change or stay the same throughout the years or if the themes change over time. If the themes do change please can you advise what they are. Please disregard any responses in Column B which suggest Nothing to improve, Nil or contain a comment which is actually a Positive comment. Thank you.”

### Prompt v2

Thank you. I have removed all the "Nil", "N/A" and "nothing" comments from the Development columns. Please can you reanalyse looking for overarching themes and describe how the change through the years. For each theme could you give me a numerical value for how many comments contributed to that theme. Could you also tell me now many Positive and Development comments there are in total.

### Prompt v3

“The spreadsheet attached shows anonymised student course evaluation comments for a 3rd Year undergraduate MBChB teaching session in the UK. Each academic year given its own tab. There are two columns - A is Positive feedback and B is Areas for Development. Please can you analyse the feedback to see if there are any over-arching trends or themes. Please can you advise if the themes change or stay the same throughout the years or if the themes change over time. If the themes do change please can you advise what they are. I have removed any comments which suggest nothing to add. Please can you tell me how many comments have contributed to each theme. Thank you”

### Prompt v4

The spreadsheet attached shows anonymised student course evaluation comments for a 3rd Year undergraduate MBChB teaching session in the UK. Each academic year given its own tab. There are two columns - A is Positive feedback and B is Areas for Development. Please can you conduct a Conceptual Content Analysis of the feedback to see if there are any over-arching trends or themes. Please can you advise if the themes change or stay the same throughout the years or if the themes change over time. If the themes do change please can you advise what they are. I have removed any comments which suggest nothing to add. Please can you tell me how many comments have contributed to each theme, both as numerical data and as a percentage. Finally please can you provide at least 5 example quotes for each theme and sub-theme, and indicate to me where you have found those quotes. Thank you

### Prompt v5

The spreadsheet attached shows anonymised student course evaluation comments for a 3rd Year undergraduate MBChB teaching session in the UK. Each academic year given its own tab. There are two columns - A is Positive feedback and B is Areas for Development. Please can you conduct a Conceptual Content Analysis of the feedback to see if there are any over-arching trends or themes. Please can you advise if the themes change or stay the same throughout the years or if the themes change over time. If the themes do change please can you advise what the changes are. I have removed any comments which suggest nothing to add. Please can you tell me how many comments have contributed to each theme, both as numerical data and as a percentage. Please can you give 5 comments as examples for each of the main and sub-themes, show me where you found them and show your evidence for using them as example quotes. Please can you use "Think Deeper" mode when you conduct this analysis. Please can you explain why your analysis may be wrong. Thank you

## ECD (MBChB Year 1; adapted from CD prompts above)

### Prompt v1

“The spreadsheet attached shows anonymised student course evaluation comments for a 1st Year undergraduate MBChB teaching session in the UK. Each academic year given its own tab. There are two columns - A is Positive feedback and B is Areas for Development. Please can you analyse the feedback to see if there are any over-arching trends or themes. Please can you advise if the themes change or stay the same throughout the years or if the themes change over time. If the themes do change please can you advise what they are. I have removed any comments which suggest nothing to add. Please can you tell me how many comments have contributed to each theme. Thank you”

### Prompt v2

The spreadsheet attached shows anonymised student course evaluation comments for a 1st Year undergraduate MBChB teaching session in the UK. Each academic year given its own tab. In 2021-22 and 2022-23 there is only one column for feedback, so comments may be positive, development areas or otherwise. In other years there are two columns - A is Positive feedback and B is Areas for Development. Please can you conduct a Conceptual Content Analysis of the feedback to see if there are any over-arching trends or themes. Please can you advise if the themes change or stay the same throughout the years or if the themes change over time. If the themes do change please can you advise what the changes are. I have removed any comments which suggest nothing to add. Please can you tell me how many comments have contributed to each theme, both as numerical data and as a percentage. Please can you give 5 comments as examples for each of the main and sub-themes, show me where you found them and show your evidence for using them as example quotes. Please can you use "Think Deeper" mode when you conduct this analysis. Please can you explain why your analysis may be wrong. Thank you

## ACD (MBChB Year 5; adapted from CD prompts above)

### Prompt v1

The spreadsheet attached shows anonymised student course evaluation comments for a 5th (final) Year undergraduate MBChB teaching session in the UK. Each academic year given its own tab. Columns A always contains Positive feedback, Column B is always Areas for Development. In some years Column C asks whether students prefer Online or Face to face teaching. In some years Column D asks the impact of online teaching on students' learning. In some years Colum E asks how the sessions have impacted on students' future career aspirations. Please can you analyse the feedback to see if there are any over-arching trends or themes. Please can you advise if the themes change or stay the same throughout the years or if the themes change over time. If the themes do change please can you advise what they are. I have removed any comments which suggest nothing to add. Please can you tell me how many comments have contributed to each theme. Thank you

# Appendix 3. Number of Responses for Year Level per Academic Year

Number and proportion of clinical debrief responses from each year level per academic year.

| Academic Year | Year 1 (ECD) | Year 3 (CD) | Year 5 (ACD) |
| --- | --- | --- | --- |
| 2016/2017 | - | 547 / 862 (63%) | - |
| 2017/2018 | - | 642 / 866 (74%) | - |
| 2018/2019 | - | 666 / 864 (77%) | - |
| 2019/2020 | - | 649 / 802 (81%) | - |
| 2020/2021 | - | 647 / 890 (73%) | - |
| 2021/2022 | 224 / 498 (45%) | 550 / 984 (56%) | 298 / 449 (66%) |
| 2022/2023 | 112 / 435 (26%) | 747 / 990 (75%) | 205 / 447 (46%) |
| 2023/2024 | 861 / 958 (90%) | 837 /1,050 (80%) | 70 / 519 (13%) |
| 2024/2025 | 215 / 496 (43%) | 738 / 960 (77%) | 118 / 526 (22%) |
| Total responses | 1,412 / 2,387 (59%) | 6,023 / 8,268 (73%) | 691 / 1,941 (36%) |

ACD; advanced clinical debrief. CD; clinical debrief. ECD; early clinical debrief. Where the total number of possible responses per year level per academic year is less than 550, this indicates where responses were only collected for one of the two semester for that academic year. Numbers higher than this indicate that responses were collected for both semesters. Where response rates are low (<40%) this reflects the approach used to engage with students, specifically whether or not the invitation to participate in the survey is distributed at the end of the last sessions for that semester, or is emailed to the student cohort after the session was finished, which noticeably affects response rates.
